# A rare case of basilar artery dissection

**DOI:** 10.3934/Neuroscience.2023008

**Published:** 2023-05-08

**Authors:** Sahibjot Bhatia, Nimrit Gahoonia, Jeffrey Stenger, Forshing Lui

**Affiliations:** 1 Clinical Sciences, California Northstate University College of Medicine, Elk Grove, CA, USA; 2 Clinical Sciences, Touro University College of Osteopathic Medicine, Vallejo, CA, USA; 3 Hospital Medicine, Kaiser Sacramento Medical Center, Sacramento, CA, USA

**Keywords:** headache, antiplatelet, anticoagulation, exercise-related headache, basilar artery dissection

## Abstract

This case describes a 30-year-old Hispanic male who presented with a significant headache that started after a period of weightlifting and squatting. The patient was diagnosed with a basilar artery dissection. His only complaint was a headache that was exacerbated with exertion and sexual activity; there were no neurologic deficits. The diagnosis of basilar artery dissection was established and supported by findings on the CT angiogram of his head and neck. Basilar artery dissections are rarely seen, as they are likely underrecognized due to their varying clinical presentations; however, it is important to consider these phenomena due to the risk of progression and high morbidity rates.

## 1. Introduction

Although there have been many reported cases of vertebral artery and carotid artery dissections, which are now considered the most common cause of stroke in young individuals [1], little is known about basilar artery dissections (BADs). BAD is an uncommon diagnosis in the general population, with its occurrence estimated to be 0.25 per 100,000 person-year [1]. Current literature suggests that BAD most commonly presents with subarachnoid hemorrhage and brain ischemia [1],[2] and is associated with high levels of morbidity and mortality [2]. However, little is known about how BAD presents, its clinical course, management modalities and long-term prognosis. We present a case of BAD that developed following strenuous exercise.

## 2. Case presentation

A 30-year-old Hispanic male with no past medical history presented to our emergency department (ED) with a new and severe headache that had worsened with exertion for the past 2 days. He reported lifting weights and squatting at the gym 2 days prior when he noticed a sudden onset of pain that started in his neck and radiated to the back of his head. He described the initial pain as sharp and stabbing in quality, rating it a 13/10. After approximately 15 minutes of rest, the severity of the headache subsided, but remained at a 6/10 even at rest. The quality or severity of the headache was not affected by posture. Prior to coming to the ED on the day of presentation, he noticed that the headache worsened to a 15/10 during sexual intercourse and while straining with defecation, which prompted him to seek medical attention. At the time of interview, he rated the headache as a 3/10. He did not experience any associated neurologic deficits or vision changes. He denied any recent trauma, falls or a loss of consciousness. The patient denied any family history of stroke, subarachnoid hemorrhage or brain aneurysms. He also denied any known history of polycystic kidney disease. The patient participated in occasional alcohol use in social settings only, but denied any history of drug use. He also denied any current use of daily medications, vitamins or supplements.

The patient's height was recorded as 1.778 m and he had a weight of 92.5 kg at the time of the encounter. He presented with stable vitals and a temperature of 36.9 °C, blood pressure of 128/85, heart rate of 88 bpm, respiratory rate of 18 breaths per minute and an oxygen saturation of 98% in the ambient air. On examination, the patient appeared comfortable and in no acute distress. His head appeared atraumatic, his heart rate and rhythm were regular and breath sounds were clear bilaterally. Cranial nerve exams were all normal, strength was 5/5 in upper and lower extremities bilaterally and sensation was intact in all extremities as well. Deep tendon reflexes were 2+/4 at the biceps, brachioradialis, triceps, patella and Achilles. NIH Stroke Scale was 0.

**Figure 1.**
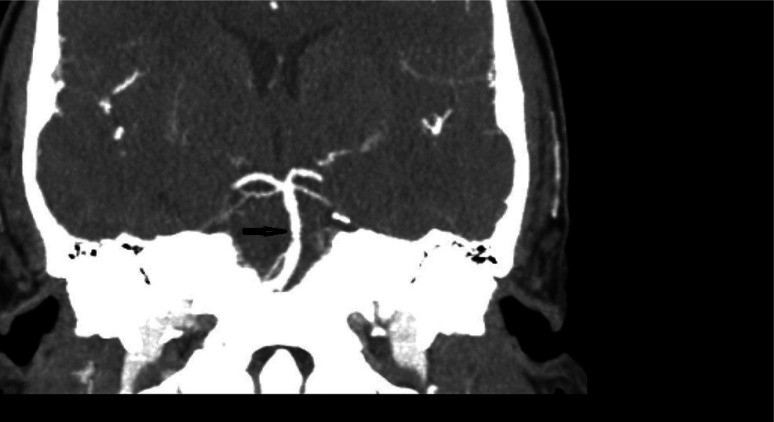
CTA coronal section showing midbasilar stenosis due to dissection.

**Figure 2.**
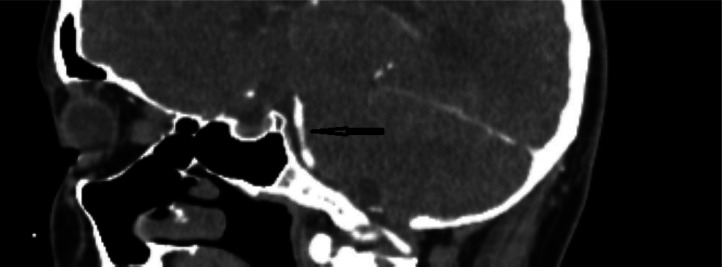
CTA sagittal section showing midbasilar stenosis due to dissection.

**Figure 3.**
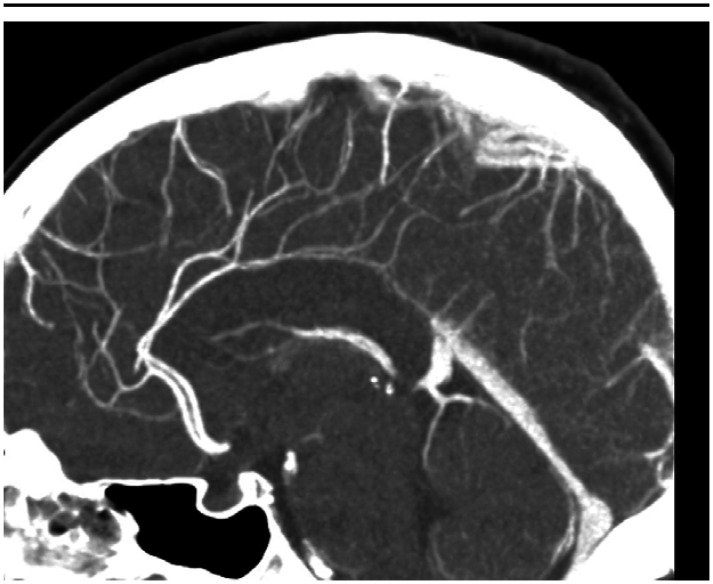
CTA showing normal cerebral arteries.

In the ED, the patient received a head computed tomography (CT) scan without contrast, which showed no acute intracranial process. A lumbar puncture was performed, revealing normal cerebral spinal fluid without blood, xanthochromia and no evidence for any infectious process. A CT angiogram (CTA) of the head and neck (Figures 1, 2) showed a long segment of moderate stenosis in the mid-basilar artery, with a sudden decrease in caliber by 50% over a 9-mm length. There were no other acute intracranial findings, and the stenosis was limited to the basilar artery. The finding was concluded by our neuroradiologist as typical for an acute BAD. At this point in the case, the neurologist did not recommend any further imaging studies, such as digital subtraction angiography (DSA) or magnetic resonance angiography (MRA), as the diagnosis of BAD was supported by the CTA findings. Of note, the CTA was also used to rule out reversible cerebral vasoconstriction syndrome, as there was no evidence of cerebral vasoconstriction, as shown in Figure 3.

The patient was started on dual antiplatelet therapy with 81 mg of aspirin and 75 mg of clopidogrel daily, and he was instructed to continue this regimen for 3 months. After this period, he was instructed to continue to take 81 mg of aspirin daily. He was also counseled on the importance of making the necessary lifestyle modifications to prevent hypertension, hyperlipidemia and diabetes (all factors that would increase the risk of dissection recurrence).

**Figure 4.**
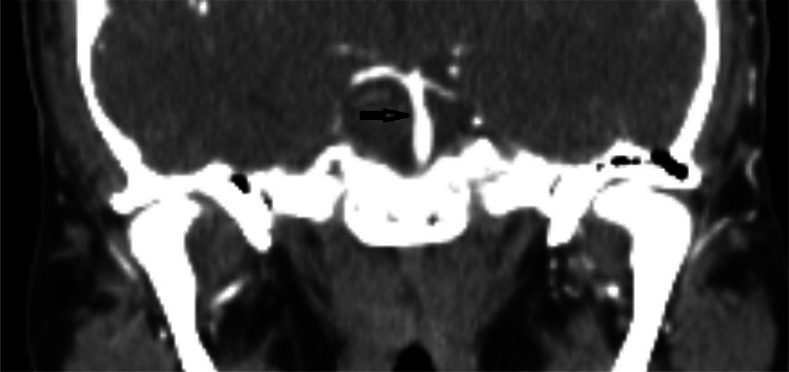
CTA coronal section 24 days post-diagnosis.

**Figure 5.**
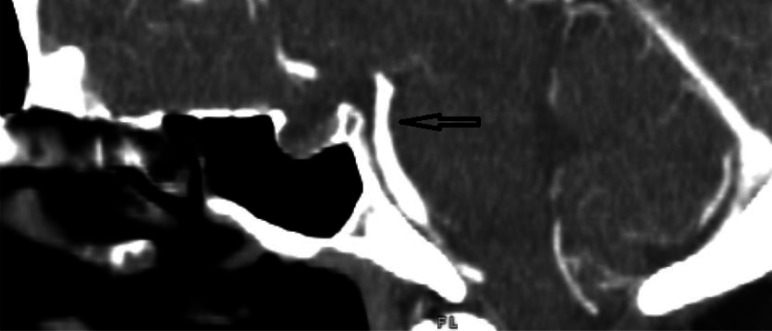
CTA sagittal section 24 days post-diagnosis.

Two weeks after discharge, the patient followed up with his neurologist outpatient and was stable and doing well. Ten days later, however, the patient complained of intermittent posterior right-sided headaches that seemed to improve with rest, sleep and application of ice to the area. At this point, his neurologist recommended that he visit the ED, where a CTA was conducted and showed improvement in the originally seen BAD without any evidence of progression (Figure 4, 5). He was discharged and his symptoms improved without intervention.

**Figure 6.**
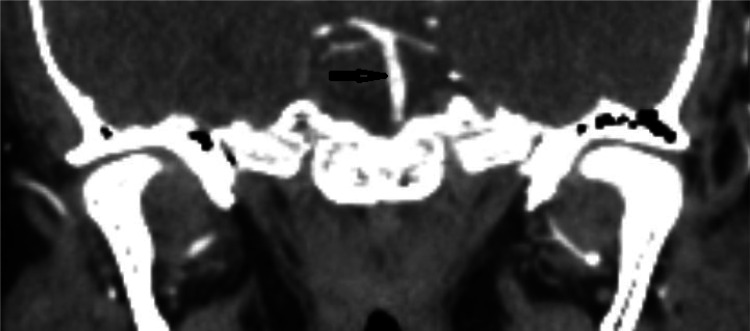
CTA coronal section taken at 3-months post-diagnosis.

**Figure 7.**
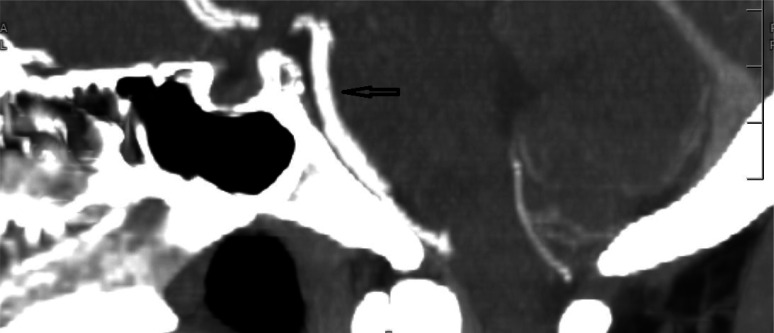
CTA sagittal section taken at 3-months post-diagnosis.

At his 3-month post-discharge visit, the patient had a repeat CTA, as originally planned, which showed no significant residual irregularity or narrowing of the basilar artery (Figure 6, 7). Good resolution of the initially seen stenosis without a need for surgery also supports the initial diagnosis of BAD. He remained stable, and clopidogrel was discontinued at this visit because the patient had completed the 3-month course.

## 3. Discussion

An arterial dissection occurs when the interior wall of an artery tears and forms a “false lumen”, which creates a new path for blood to flow into and become static within a pouch [3]. This blood can begin to form clots, which can cause a stroke by blocking the flow in a true artery or cause a distal territory ischemic infraction via an artery-to-artery embolism [3]. Predisposing factors to an arterial dissection include, but are not limited to, a connective tissue disorder, fibromuscular dysplasia, smoking, hypertension and cystic medial necrosis [4],[5]. Although arterial dissections are considered one of the leading causes of ischemic stroke in patients younger than 45, the carotid and vertebral arteries are most commonly involved; the basilar artery is typically not affected [6]. Typical presentation of a BAD includes symptoms caused by a brainstem or cerebellar ischemic stroke, but it can also present with a subarachnoid hemorrhage [6].

With BADs being rare themselves, this case was an atypical presentation for many reasons. Not only was there an absence of any known preexisting risk factors for dissection, but the patient's only presenting symptom was a significant headache that was aggravated with sex and exertion without any focal neurologic deficits. The most probable cause for this patient's BAD was likely exercise and exertion. In fact, a previous case report has suggested that nearly 40% of spontaneous cerebrovascular dissections are correlated with cervical trauma induced secondary to exercise [7]. Exercise- or sexual activity-associated headaches have been reported as presenting symptoms of arterial dissections [8],[9]. One specific paper stresses the importance of considering BAD in patients with coital headaches, as it reports on a patient who presented with a sudden-onset thunderclap headache, where the patient was suspected to have a subarachnoid hemorrhage but was diagnosed with BAD based on MRA [10].

The initial leading diagnosis due to the nature of this patient's presentation in this case was a subarachnoid hemorrhage; however, this was quickly ruled out after performing a non-contrast head CT and lumbar puncture. Headache secondary to drug use was also considered; however, the patient denied any history of drug use. Furthermore, drug use-induced headaches are unlikely to present with such high severity. Given the patient's severity of headaches, an intracranial process was highly suspected to be the cause of the patient's presentation. Therefore, a CT angiogram of the head and neck (Figure 1, 2) was obtained and showed an irregular contour and abrupt narrowing of the mid-basilar artery, which allowed for the diagnosis of BAD. Reversible cerebral vasoconstriction syndrome was also considered as a potential diagnosis; however, it was ruled out based on there being no evidence of vasoconstriction in the cerebral vessels (Figure 3). Although a digital subtraction angiography is considered the best diagnostic tool for a cranial dissection [11], CT and CT angiography are considered the first line of investigation and were chosen because they are noninvasive and efficient imaging tools which are widely used (along with MRA) for diagnosis. In this specific case, the neurologist did not believe any further imaging beyond the CTA was required, as the suspected diagnosis of acute BAD was supported by the imaging findings. A follow-up DSA or MRA would further support this diagnosis; however, it was not completed during this visit. Furthermore, good resolution of the stenosis, as seen in follow-up images without surgery, also supported the diagnosis of BAD.

There are no clear guidelines for treating cerebrovascular dissections. Generally utilized treatment for an unruptured BAD involves anticoagulation or antiplatelet therapy with strict blood pressure control, with a possibility of stent placement, depending on the severity of symptoms. In a study of 11 patients with unruptured BAD, nine were treated with anticoagulation therapy and two required stent placement. Of these nine, four patients had signs of progressive ischemia, while the other five had no signs of further disease progression [12]. Due to the lack of any specific guidance for treating intracranial and extracranial artery dissections, the European Stroke Organization conducted a literature review analysis in an attempt to establish guidelines. They found that there was no significant difference in outcomes or adverse effects when utilizing anticoagulant versus antiplatelet medications to treat cerebrovascular dissections [13]. Another study, in which 55% of patients with spontaneous dissection received antiplatelet medication, 29.4% received anticoagulation and 12.6% received combined treatment, there was no significant difference in the rate of new or recurrent events when all treatment choices were compared [14]. The treatment modality chosen in this case involved the use of dual antiplatelet medications for 3 months, followed by continuous use of a single antiplatelet medication for life. The neurologist involved in this case recommended this treatment based on the lack of risk factors in this patient and a relatively high risk of the dissection progressing to brain ischemia.

Overall, since BAD is a rare condition with variable clinical presentations, it is likely that many cases are underdiagnosed. Previous case reports have shown that BAD typically presents along with a subarachnoid hemorrhage [1]. Although this patient's severe headache would point to a similar diagnosis, the imaging displayed only an isolated BAD. Thus, this case report represents a clear deviation from the initial differential diagnoses typically suspected. It is important for providers to recognize nonspecific symptoms such as exertional or post-coital headaches that start occurring after a seemingly harmless event, and for them to consider a cerebrovascular dissection as a potential diagnosis.

## 4. Conclusion

BAD is a rare diagnosis. It can present with seemingly nonspecific and varying symptoms which may not always provoke an immediate clinical suspicion of a dissection. In this case report, we present a symptomatic clinical presentation of BAD that is often underrecognized. The primary motivation behind presenting this case was to bring awareness to an emergency situation that can quickly progress to irreversible, lifelong neurologic deficits if unrecognized.
